# Retracted: Effects of Scrambler Therapy in Patients with Failed Back Surgery Syndromes and Factors Associated with Depression Affecting Pain before and after the Therapy

**DOI:** 10.1155/2021/2572391

**Published:** 2021-01-23

**Authors:** Pain Research and Management

*Pain Research and Management* has retracted the article titled “Effects of Scrambler Therapy in Patients with Failed Back Surgery Syndromes and Factors Associated with Depression Affecting Pain before and after the Therapy” [1]. Scrambler Therapy® is a registered trademark held by Prof. Giuseppe Marineo and may only be used to refer to approved medical devices, which does not include the Pain Jammer ENS-1140.

The authors do not agree with retraction.
